# 

**Published:** 1890-08

**Authors:** 


CONTENTS:

PAGE

ORIGINAL ARTICLES:

Some Original Investigations upon the Python Molurus. By Leffingwell Hatch, B.S., M.D . . . . 415

Texas Fever (Southern Cattle Fever, Etc.). By Paul Paquin . . . . 436

Age of the Horse, Ox, Dog, and other Domesticated Animals. By R. S. Huidekoper, M.D., Veterinarian . . . . 455

Prolapsus and Amputation of the Rectum. By James A. Waugh, Veterinarian, Sixth Cavalry, U. S. A . . . . 462

EDITORIAL:

Veterinary Journalism . . . . 464

U. S. Army Veterinary Notes . . . . 466

United States Veterinary Medical Association . . . . 466

SOCIETY PROCEEDINGS:

Regular Meeting of the Massachusetts Veterinary Association . . . . 467

VETERINARY COLLEGE NOTES:

University of State of Ohio . . . . 476

CORRESPONDENCE:

Reply to Open Letter . . . . 476

NECROLOGY:

Armand Charles Goubaux . . . . 479

REVIEW DEPARTMENT . . . . 480

BOOKS AND PAMPHLETS RECEIVED . . . . 482

